# Iron and late preterm

**DOI:** 10.1186/1824-7288-40-S2-A39

**Published:** 2014-10-09

**Authors:** Rita Luciano

**Affiliations:** 1 Neonatology Unit, Gemelli University Hospital, 00169 Rome, Italy

## 

Iron is essential for the Central Nervous System development, i.e. mielinization process and cellular differentiation as well as correct functioning of neurotransmitters [1]. Preterm infants show an increased risk of iron deficiency (ID) since 80% of the iron storage at birth is accumulated during the third trimester of pregnancy. Rapid child growth and elevated red cell turn over in the neonatal period may exaust iron storage after two months of age [2]. ID risk is greater in the breast-fed babies, since maternal milk does not contain an amount of iron sufficient to demands. However, ID prevalence equal to 14%, between the fourth and the eighth month of age [3], is also reported in the preterm infants who are nourished with milk formulas enriched with iron. Referred long-lasting effects of ID in infancy include reduced cognitive functions, motor performances and social-emotional development, as well as persisting neurophysiologic abnormalities [1]. As a consequence early iron supplementation is recommended for preterm and very-low-birth-weight infants [3]. Healthy late-preterm infants are often treated with the same modalities than term neonates. For these infants we lack strong evidence based recommendations about supplementation of iron, doses, time of beginning as well as duration of treatment. The RCT evidence to date does not suggest a definite threshold of birth-weight or gestational age at which iron supplementation becomes beneficial. Two methodologically sound trials suggest a benefit even for marginally low-birth-weight infants, whether term or preterm [4]. As recently reported, marginally low-birth-weight neonates showed a high prevalence of ID and Iron Deficiency Anemia (IDA) when evaluated at the age of six months, especially in the case of exclusive breast feeding to age six weeks [5]. Martial supplementation lowered ID and IDA prevalence without adverse effects. In conclusion, despite the amount of studies concerning ID in infancy, there is still a paucity of evidence about the effects of iron deficiency/overload with respect to growth , morbidity and neurodevelopmental outcomes in the different categories of neonates ,as well as about the reliability of the presently available iron methabolism markers.
